# Using retinal electrophysiology toward precision psychiatry

**DOI:** 10.1192/j.eurpsy.2022.3

**Published:** 2022-01-14

**Authors:** Thomas Schwitzer, Marion Leboyer, Vincent Laprévote, Valérie Louis Dorr, Raymund Schwan

**Affiliations:** 1 Pôle Hospitalo-Universitaire de Psychiatrie d’Adultes et d’Addictologie du Grand Nancy, Centre Psychothérapique de Nancy, Laxou, France; 2 IADI, INSERM U1254, Université de Lorraine, Nancy, France; 3 Faculté de Médecine, Université de Lorraine, Vandœuvre-lès-Nancy, France; 4 Fondation FondaMental, Créteil, France; 5 Université Paris Est Creteil (UPEC), AP-HP, Hôpitaux Universitaires “H. Mondor”, DMU IMPACT, FHU ADAPT, INSERM U955, IMRB, Translational Neuropsychiatry laboratory, F-94010 Creteil, France; 6 INSERM U1114, Fédération de Médecine Translationnelle de Strasbourg, Département de Psychiatrie, Centre Hospitalier Régional Universitaire de Strasbourg, Strasbourg, France; 7 CRAN, Université de Lorraine, CNRS, Nancy, France

**Keywords:** Biomarkers, electrophysiology, precision medicine, retina

## Abstract

Precision medicine in psychiatry is based on the identification of homogeneous subgroups of patients with the help of biosignatures—sets of biomarkers—in order to enhance diagnosis, stratification of patients, prognosis, evaluation, and prediction of treatment response. Within the broad domain of biomarker discovery, we propose retinal electrophysiology as a tool for identification of biosignatures. The retina is a window to the brain and provides an indirect access to brain functioning in psychiatric disorders. The retina is organized in layers of specialized neurons which share similar functional properties with brain neurons. The functioning of these neurons can be evaluated by electrophysiological techniques named electroretinogram (ERG). Since the study of retinal functioning gives a unique opportunity to have an indirect access to brain neurons, retinal dysfunctions observed in psychiatric disorders inform on brain abnormalities. Up to now, retinal dysfunctions observed in psychiatric disorders provide indicators for diagnosis, identification of subgroups of patients, prognosis, evaluation, and prediction of treatment response. The use of signal processing and machine learning applied on ERG data enhances retinal markers extraction, thus providing robust, reproducible, and reliable retinal electrophysiological markers to identify biosignatures in precision psychiatry. We propose that retinal electrophysiology may be considered as a new approach in the domain of electrophysiology and could now be added to the routine evaluations in psychiatric disorders. Retinal electrophysiology may provide, in combination with other approaches and techniques, sets of biomarkers to produce biosignatures in mental health.

Psychiatry is the last medical field almost exclusively based on subjective clinical evaluation without the help of quantitative biomarkers for diagnosis, stratification and classification of patients, prognosis, evaluation, and prediction of treatment response and detection of subjects at high risk of psychiatric disorders [1]. This is holding back the development of precision medicine in psychiatry, which is based on the identification of homogeneous subgroups of patients with the help of biosignatures in order to enhance diagnosis, classification, prognosis, and interventions [1]. To plan the future, we need to revise health care organization which will be based on the use of biological markers based on diverse approaches in order to produce biosignatures with the help of signal biology and computational psychiatry [1]. Within the broad domain of biomarker discovery, we support that retinal electrophysiology is a promising tool for the identification of biosignatures. Here, we describe how retinal electrophysiology based on electroretinogram (ERG) measurements could be: (a) collected as routine measures for patients; (b) analyzed with robust pre- and post-signal processing (mathematical and machine learning approaches); (c) added to other collected data in patients’ medical record; and (d) added to the sets of biomarkers to produce biosignatures for precision medicine in psychiatry.

The retina is considered as a window to the brain, thus offering an indirect access to the functioning brain in psychiatric disorders [2]. The retina is a complex neural network organized in layers of specific retinal neurons [3]. These neurons share similar anatomical and functional properties with brain neurons [3]. The functional properties of these neurons can be studied by retinal electrophysiological techniques named ERG [4]. Using flashes, checkerboards, and hexagons stimulation for flash ERG (fERG), pattern ERG (PERG), and multifocal ERG (mfERG), respectively, ERGs allow the evaluation of the functioning of each neuron of the retina [4]. ERGs give objective, reliable, easy-to-use, and highly reproducible measures with a low number of repetitive stimulations [4]. From the functioning of each of these neurons, different pathophysiological processes can be identified, giving various endophenotypes [2]. Since retinal neurons give a unique opportunity to have an indirect access to brain neurons, retinal dysfunctions observed in psychiatric disorders inform on brain abnormalities [2]. Retinal dysfunctions as measured with ERGs are observed in many psychiatric disorders such as major depressive disorders (MDDs), bipolar disorders (BDs), schizophrenia (SZ), autism spectrum disorders, seasonal affective disorders, and addictive disorders, to name a few [5]. These data indicate that retinal neurons are sensitive to various pathophysiological mechanisms in the central nervous system and involved in psychiatric disorders—neurotransmission dysfunctions, inflammation, neurodegeneration, and autoimmunity [2]—thus giving information on specific endophenotypes. Up to now, retinal dysfunctions were often described in patients compared to healthy subjects [5]. Interestingly, recent studies also compare retinal parameters between pathologies [5]. Available data support the use of retinal electrophysiology as biomarkers for the identification of biosignatures to produce better diagnosis, stratification, prognosis, and treatment. By coupling several ERG recordings—fERG and PERG—patients with MDD and BD may be differentiated, since they show different retinal responses and have specific retinal dysfunctions [5]. This could serve as a crucial assessment tool in the context of a first depressive episode in order to know whether it is a bipolar or unipolar depressive disorder. Retinal electrophysiology may serve for stratification and identification of subgroups of patients. We showed that patients with SZ having visual hallucinations have dysfunctions of the rod system—as showed by an increased in rod b-wave implicit time of the dark-adapted 0.01 fERG—compared to patients without visual hallucinations and to control group [6]. In MDD, specific links between clinical features—anhedonia, suicidal ideations, and melancholia—and ERG parameters were described [5]. Retinal measures may be useful for the detection of subgroups of subjects at high risk for mental disorders [5]. In subjects at high risk for SZ or BD, specific retinal dysfunctions—as showed by altered rod Vmax b-wave amplitude and implicit time of the fERG—were found [5]. Thus, the retinal function evaluated by fERG may mark neurodevelopmental pathways and thus give early preclinical traits in high-risk subjects for psychiatric disorders [5]. This is particularly critical for the surveillance and follow-up of at-risk subjects. Since cannabis use can impact the transition to a psychiatric disorder (SZ or BD) in young people, it is also crucial to consider retinal biomarkers of cannabis use. Interestingly, we showed several retinal dysfunctions in cannabis users using the fERG, the PERG, the mfERG, and the On–Off ERG, which may reflect the impact of regular cannabis use on central dopaminergic and glutamatergic neurotransmission [7]. Most importantly, both similar and different retinal dysfunctions were observed in cannabis users and SZ patients [5]. Future studies will validate whether retinal electrophysiology could serve as biomarkers of a transition to a psychiatric disorder in cannabis users. Finally, retinal electrophysiology may help to assess prognosis. In offspring having psychotic-like experiences, the cone Vmax implicit time of the fERG was increased and was associated with a lower global IQ and with deteriorated global functioning [8]. This suggests that ERG parameters could be linked to the prognosis. Retinal electrophysiology may also be used to monitor and predict the treatment response as well as to distinguish responders and nonresponders to psychotropic drugs [5]. For example, b-wave amplitude of the fERG was found to be altered differentially by antidepressants in responders and nonresponders MDD patients [5]. Using retinal sensitivity, defined by the recording of retinal functioning by fERG following several flashes stimulations with increasing intensities, medicated and drug free major depressed patients had different retinal responses [5], suggesting specific retinal markers of treatment. Based on these results, future studies will also highlight the relevance of retinal electrophysiology in detecting patients with resistant pathologies, since these are clinical situations for which we need biosignatures.

The use of signal processing and machine learning tools applied to retinal measurements represents a crucial step in precision psychiatry. Extracting the raw signal ensures an access to all relevant information derived from retinal signal. Analysis of retinal electrophysiological signals is mainly based on amplitude and peak time extracted from waves of interest (mainly a- and b-waves for the fERG and P50 and N95 for the PERG) [4]. Signal processing applied to ERG allows to isolate many other interesting and relevant characteristics from raw data. Since retinal signal may be quite low (few microvolt to millivolt), it can be perturbed by endogenous electrophysiological perturbations sources such as deep brain sources, muscle sources, or energy network. The instrumentation chain ensures the amplification and prefiltering of the signal in the adapted band of the signal before the acquisition. However, this is sometimes insufficient for high-quality ERG. Thus, it is necessary to preprocess the digital data before extracting retinal electrophysiological markers. Specific signal processing may be applied according to each of ERG acquisition. Using more advanced machine learning algorithms can also be applied on ERG signals to enhance retinal markers extraction. The future steps allowed by these techniques are to improve ERG signal quality, wave detection, and identification of retinal electrophysiological markers from ERG recordings. By coupling conventional and machine learning approaches, ERG can give robust, reproducible, and reliable retinal electrophysiological markers to produce biosignatures in precision psychiatry.

We argue here that retinal electrophysiology may be considered as a new approach in the domain of electrophysiology and may provide, in combination with other approaches and techniques, a set of biomarkers to produce biosignatures in mental health. To this end, we need to consider all collected data for each patient. Interestingly, in routine care, more relevant information is already collected by clinicians such as clinical data, biology, neuropsychology, imaging, and electrophysiology. We stress that retinal electrophysiology should now be added to these routine evaluations in mental health to produce biosignatures (Figure 1). To consider all these collected data, the development of systems biology and computational psychiatry based on artificial intelligence techniques is crucial. Two complementary approaches can be considered for these developments: (a) clinical trials allow controlled environments in which clinical characteristics of patients are well known: diagnosis, medication, comorbidity, or not, to name a few and (b) routine care allows gathering of real-word data associated with medical observations and standard questionnaires. Routine care can be fed with predictions from digital twins and assist psychiatrists for better care while continuously improving the numerical models from each patient inclusion.

**Figure 1.**
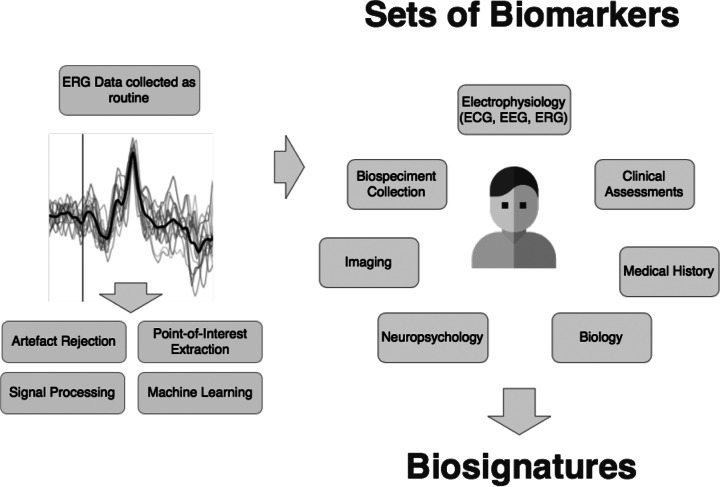
Retinal electrophysiology added in sets of biomarkers to identify biosignatures in mental health. Retinal electrophysiology as measured with ERG and collected as routine measures for patients. Signal processing and machine learning techniques applied on ERG signal. ERG integrated in sets of biomarkers in combination with other approaches and domains to identify biosignatures in mental health. *Abbreviations*: ECG, electrocardiography; EEG, electroencephalography; ERG, electroretinogram.

Retinal electrophysiology shows great potential for objective assessments in psychiatric disorders and should be studied in various pathological conditions and subgroups of patients with highly reproducible and standardized protocols and measurements. To develop relevant retinal electrophysiological biomarkers, they must be available for routine clinical practices with small footprint, cost-effective, rapid and easy to perform, and highly reproducible. Interestingly, portable and connected ERG devices are available today and can feed routine clinical evaluation. These new retinal devices are easy to use, connected, and secured with semiautonomous remote analysis with interpretations validated by a medical expert ensuring reproducibility and reliability. This chain analysis is particularly adapted for identification and development of biomarker signatures in mental health.

To conclude, retinal electrophysiology is a promising instrument for developing signal processing and machine learning tools that should inform clinical decision for better diagnosis, classification, prognosis, treatment, and detection of high-risk subjects.
